# Effect of length of time spent on the receiving diet on growth performance, rumination activity, and carcass outcomes in beef × dairy steers

**DOI:** 10.1093/tas/txag151

**Published:** 2026-10-03

**Authors:** Becca G Francis, Forest L Francis, Ana Clara Baiao Menezes, Tony C Bryant, Warren C Rusche, Zachary K Smith

**Affiliations:** Department of Animal Science, South Dakota State University, Brookings, SD 57007, United States; Department of Animal Science, South Dakota State University, Brookings, SD 57007, United States; Department of Animal Science, South Dakota State University, Brookings, SD 57007, United States; Five Rivers Cattle Feeding, Johnstown, CO 80534, United States; Department of Animal Science, South Dakota State University, Brookings, SD 57007, United States; Department of Animal Science, South Dakota State University, Brookings, SD 57007, United States

**Keywords:** Angus × Holstein, liver abscess, receiving diet, rumination

## Abstract

We investigated the effect of increasing length of time spent on a 30% roughage equivalent (RE) receiving diet on growth performance, carcass characteristics, rumination activity, and serum metabolites in Angus × Holstein steers. Angus × Holstein steers (*n* = 144) were allotted by BW 18 pens (*n* = 8 steers/pen) in a randomized complete block design. A corn silage-based receiving diet, formulated to provide 30% roughage equivalency (RE; 60% DM basis) was fed for either 28 (REC28), 56 (REC56), or 84 d (REC84) post-feedlot arrival followed by a 12% RE finishing diet (6 replicate pens per treatment). Dry matter intake was reduced (*P* = 0.10) with increasing time spent on the receiving diet during the first 97 DOF. This reflected the reduced ADG and BW observed of the REC84 steers on d 97 (*P* < 0.01). No differences (*P* ≥ 0.18) were observed for post-receiving period ADG, DMI, and G:F. Less fat thickness (*P* = 0.07) was observed for REC84 steers, but no other differences (*P* ≥ 0.19) in carcass characteristics among treatments were noted. A numerical reduction in total liver abscessation was observed, driven by a numeric reduction (*P* = 0.13) in minor abscesses with increasing time spent on a 30% RE receiving diet. Increased time spent on the receiving ration was positively associated with rumination activity (*P* < 0.01). However, rumination activity (min/kg of NDF and peNDF consumed) decreased (*P* ≤ 0.02) with increasing time spent on the receiving diet. Dietary treatments did not affect (*P* ≥ 0.18) circulating concentrations of serum NEFA or glucose. Steers on the REC84 treatment exhibited the greatest concentration of circulating SUN (12.76 mg/dL), followed by steers on the REC28 treatment (12.41 mg/dL), and steers on the REC56 treatment had the least concentration of circulating SUN (11.49 mg/dL). Greater time spent on the receiving ration resulted in reduced DMI, ADG, and BW during the first 97 d, but did not alter overall feeding performance or carcass characteristics, including prevalence of liver abscesses. Increased time spent on the receiving diet positively influenced absolute rumination activity and minimally affected circulating concentrations of serum metabolites.

## Teaser

Increased time spent on the receiving diet positively influenced absolute rumination activity and minimally affected circulating concentrations of sera metabolites.

## Introduction

Dairy-influenced cattle contribute to the U.S. fed-beef supply, accounting for 16.3% of the supply in 2016 (Boykin et al. 2017). Within dairy-influenced feeder calves, a noticeable shift has occurred, favoring beef × dairy crossbreeding over straightbred Holstein genetics. Historically, dairy-influenced steers have provided a consistent, year-round supply of feeder calves, thus contributing to the fed beef system when cattle availability is limited (Zinn et al. 2016; McCabe et al. 2022). Multiple interrelated factors, including strategic use of sexed semen (De Vries et al. 2008; Seidel and DeJarnette 2022), improved reproductive management (Berry et al. 2014), economic incentives (Basiel and Felix 2022), and a focus on increasing Holstein steer value (McCabe et al. 2022), have contributed to this shift towards beef × dairy crossbreeding by dairies. Recent experiments have confirmed the improved performance of beef × dairy genetics compared to straightbred Holsteins (Foraker et al. 2022; Carvalho et al. 2024; Pimentel-Concepción et al. 2024). Fed-Holsteins in the High Plains are associated with greater prevalence of liver abscesses compared to beef steers, likely because of their greater lifetime consumption of low-roughage, concentrate-based diets, creating increased risk for ruminal acidosis (Amachawadi and Nagaraja 2016) and sensitivity to weather changes.

Receiving rations offered to newly-arrived feeder calves are high-forage (>30% DM inclusion; Samuelson et al. 2016). For beef feeder calves, the purpose of the receiving ration is to: (1) stimulate intake by offering a diet that is similar in characteristics to what was previously consumed and (2) begin adaptation of the ruminal microbes to a high-energy, concentrate-based diet. However, dairy-influenced calves are offered energy-dense calf-starters from an early age (approximately 6 d old; Machado and Ballou 2022). Thus, upon arrival at the feedlot, they are already accustomed to consuming concentrate-based diets and eating feed from a bunk (Duff and McMurphy 2007). Therefore, conventional reasons for the receiving diet do not necessarily apply to them. Published literature on receiving period management of Holstein calves is limited, and currently non-existent in beef × dairy crossbreds. Although, some have reported feeding a single, grain-based finishing diet upon arrival and throughout the feeding period to Holstein steers (Zinn et al. 2007; Carvalho et al. 2024). There are certainly implications of lifetime grain consumption on rumen health and prevalence of liver abscesses in dairy-influenced fed cattle. Regardless, greater inclusions of both roughage and roughage NDF have been observed as a strategy for reducing liver abscesses in cattle (Zinn and Plascencia 1996; McDaniel et al. 2024; Word et al. 2024). However, greater roughage inclusion in diets fed to dairy-influenced steers may not be widely implemented because of detriments to early feeding-period growth performance.

We hypothesized that steers spending less time on the receiving diet, and thus spending more time on a finishing diet, would have improved growth performance, but may result in increased prevalence of liver abscesses at slaughter. Therefore, we aimed to evaluate the effect of increasing length of time spent on the receiving ration on growth performance, carcass characteristics, rumination activity, and sera metabolites of Angus × Holstein steers fed for 284 days.

## Materials and methods

This study was conducted at the Ruminant Nutrition Center in Brookings, SD between April 2024 and January 2025. The animal care and handling procedures used in this study were approved by the South Dakota State University Animal Care and Use Committee (#2412-014E).

### Cattle and feeding management

One hundred and forty-four Angus × Holstein steers [initial shrunk body weight (BW) = 192 kg ± 17.1 kg] were single-sourced from a grower yard in southwest Iowa and transported to the Ruminant Nutrition Center in Brookings, SD. Steers were previously fed a 16% crude protein complete textured calf starter consisting of whole and steam-flaked corn, protein pellets, molasses, and oats. Steers were fed 3 times daily and were reportedly consuming 3 to 4% of BW on a dry matter (DM) basis before arrival to the study site. Steers were unloaded at the study site on d -2 and placed into 15 pen with concrete floors (∼10 hd/pen). Steers were offered long-stem hay top-dressed with a total mixed ration (TMR) at 1.0% of BW on a DM basis and allowed ad libitum access to water and were left to rest overnight. The following day (d − 1), the steers were pulled from their pens for standard initial processing, where they received a visual ear tag, were vaccinated for respiratory (BoviShield Gold One Shot; Zoetis Animal Health, Parsippany, NJ) and clostridial (Ultrabac 7/Somubac; Zoetis Animal Health) diseases, and treated for internal and external parasites (Cydectin, Elanco Animal Health, Greenfield, IN). Since the previous health history of the steers was unknown, a metaphylactic dose of tulathromycin and ketoprofen (Draxxin KP; Zoetis Animal Health) was administered to all steers. An individual BW was obtained for allotment purposes and all BW measures recorded during the study were shrunk 4% to account for digestive tract fill for the calculation of growth performance. A 3-axis accelerometer Allflex SenseHub Dairy tag (SCR Engineers Ltd, Netanya, Israel) was placed in the middle one-third of the right ear of all steers and remained in place to continuously measure daily and hourly rumination time (min). This technology has been validated as a mechanism for quantifying time spent ruminating (Wolfger et al. 2015). Although every steer received a rumination tag at initial processing, it was discovered post-processing that some tags were inoperable. Therefore, 3 d after processing, tags were moved across pens to ensure equal representation of rumination tags across treatments (43 tags/treatment; 6–8 tags/pen). Data were downloaded at the conclusion of the study; the time-period steers were on the receiving diet (d 84), in addition to the 7 day transition period and 7 days on the finishing ration + 14 d (d 97) were considered for statistical evaluation (d 1 to 97) as the authors did not anticipate there to be notable differences in rumination time once all steers were on the same finishing diet (post-97 d).

Pens (*n* = 18) were allotted to one of three treatments and steers were allotted to treatment pens (*n* = 8 animals/pen). Pens were blocked by location within the feedyard. This provided six replicated pens per treatment. Treatments consisted of:

1. A 30% roughage equivalent (RE) corn silage-based receiving diet (60% DM basis) fed for 28 d (REC28).
2. A 30% roughage equivalent (RE) corn silage-based receiving diet (60% DM basis) fed for 56 d (REC56).
3. A 30% roughage equivalent (RE) corn silage-based receiving diet (60% DM basis) fed for 84 d (REC84).

The 30% RE receiving diet (Table 1) consisted of corn silage, MDGS, a molasses-based suspended supplement, and included dry-rolled corn and high-moisture corn in a 50:50 blend. Based on the analyzed starch content (starch = 35.28% DM basis), the corn silage was determined to contribute 51% roughage on a DM basis to achieve the targeted roughage equivalency in each treatment diet. After the allotted time spent on the receiving diet, steers were transitioned to a 12% RE finishing diet by offering a 21% RE diet for 7 d prior to the finishing diet (12% RE). As a result of feed ingredient availability, diet formulation changed multiple times during the 284-d feeding period following the 97-d receiving period and is reflected in Table 2. Pen fencing was constructed with pipe and cable, while the pen dimensions were 7.62 m × 7.62 m, with a 7.62 m concrete bunk-line and concrete pen floors bedded with straw. All pens were equipped with a continuous-flow heated waterer. Cattle bunk space was restricted to 30.48 cm/hd to mimic industry standards via painted lines on the inside of the feed bunks.

**Table 1:** Ingredient and nutrient composition of diets fed to Angus × Holstein steers during the receiving period (d 1–97).

Item, % DM basis	Diets^1^		
	30R	21R	12R
** Dry rolled corn**	6.36	16.68	25.08
** High moisture corn**	6.27	15.71	24.83
** MDGS^2^**	26.42	27.02	24.29
** Liquid supplement^3^**	6.41	6.36	6.00
** Corn silage**	54.54	34.23	19.81
**Nutrient composition^4^, DM basis**			
** Dry matter, %**	40.5	45.9	55.1
** Crude protein, %**	17.8	17.7	16.7
** Neutral detergent fiber, %**	34.1	27.6	22.4
** rNDF^5^**	23.9	15.0	8.7
** Acid detergent fiber, %**	18.4	14.1	10.8
** Ether extract, %**	4.4	4.6	4.6
** NE_m_, Mcal/kg**	1.85	1.97	2.06
** NE_g_, Mcal/kg**	1.23	1.32	1.40

1 30R: 30% roughage equivalent fed for either 28, 56 or 84 d; 21R: 21% roughage equivalent (fed for 7 d following the 30R diet); 12R: 12% roughage equivalent fed for the remainder of the feeding period.

2 Modified distillers grains plus solubles.

3 Liquid supplement provided (DM basis): 61.82% crude protein, 56.0% nonprotein nitrogen, 0.90 mcal/kg net energy for maintenance, 0.57 mcal/kg of net energy for gain, 1.34% crude fat, 12.0% calcium, 0.38% P, 2.50% K, 0.14% Mg, 5.0% NaCl, 3.0% S, 1.04 ppm Co, 200.0 ppm Cu, 12.0 ppm I, 15.10 mg/kg of EDDI, 469.50 ppm Fe, 400.0 ppm Mn, 3.0 ppm, Se, 1800.0 ppm Zn, 44,092.45 IU/kg of vitamin A, 440.92 IU/kg of vitamin E, and 661.52 mg/kg of monensin sodium (Rumensin, Elanco, Indianapolis, IN, USA).

4 Crude protein analyzed for all ingredients; Neutral detergent and acid detergent fiber analyzed for all ingredients except dry rolled corn and high moisture corn (tabular value of 9 and 3% assigned for neutral detergent and acid detergent fiber, respectively); Ether extract analyzed for MDGS, all other ingredients were assigned tabular values from NASEM (2016); Net energy for maintenance (NEm) and net energy for gain (NEg) were assigned from tabular values (NASEM 2016).

5 rNDF: roughage; NDF: corn silage inclusion, % × NDF, % of corn silage.

**Table 2:** Ingredient and nutrient composition of finishing diets fed to Angus × Holstein steers from d 98 to 284.

	Diets				
Item^1^, % DM basis	d 98 to 112	d 113 to 154	d 155 to 168	d 169 to 272	d 273 to 284
** Dry rolled corn**	25.29	–	–	64.22	63.95
** High moisture corn**	23.95	9.72	10.33	–	–
** High moisture ear corn (earlage)**	–	58.83	56.89	–	–
** MDGS**	24.04	25.62	–	–	–
** DDGS**	–	–	26.68	20.37	20.43
** Liquid supplement^2^**	5.93	5.83	6.10	5.27	5.28
** Rye silage**	–	–	–	10.14	–
** Corn silage**	20.78	–	–	–	–
** Sorghum/Corn silage blend**	–	–	–	–	10.34
**Nutrient composition^3^, DM basis**					
** DM, %**	55.7	59.7	66.9	75.5	77.4
** CP, %**	16.4	16.4	16.4	15.0	14.5
** NDF, %**	24.4	26.3	27.5	20.8	19.3
** ADF, %**	12.3	10.4	11.4	9.6	8.0
** NE_m_, Mcal/kg**	2.03	2.03	2.03	2.08	2.10
** NE_g_, Mcal/kg**	1.39	1.39	1.39	1.38	1.41

1 MDGS: modified distillers grains plus solubles; DDGS: dry distillers grains plus solubles.

2 Liquid supplement provided (DM basis): 61.82% crude protein, 56.0% nonprotein nitrogen, 0.90 mcal/kg net energy for maintenance, 0.57 mcal/kg of net energy for gain, 1.34% crude fat, 12.0% calcium, 0.38% P, 2.50% K, 0.14% Mg, 5.0% NaCl, 3.0% S, 1.04 ppm Co, 200.0 ppm Cu, 12.0 ppm I, 15.10 mg/kg of EDDI, 469.50 ppm Fe, 400.0 ppm Mn, 3.0 ppm, Se, 1,800.0 ppm Zn, 44,092.45 IU/kg of vitamin A, 440.92 IU/kg of vitamin E, and 661.52 mg/kg of monensin sodium (Rumensin, Elanco, Indianapolis, IN, USA).

3 Crude protein analyzed for all ingredients; Neutral detergent and acid detergent fiber analyzed for all ingredients except dry rolled corn and high moisture corn (tabular value of 9 and 3% assigned for neutral detergent and acid detergent fiber, respectively); Ether extract analyzed for MDGS and DDGS, all other ingredients were assigned tabular values from NASEM (2016); Net energy for maintenance (NEm) and net energy for gain (NEg) were assigned from tabular values (NASEM 2016).

Individual feed ingredients were sampled weekly for the duration of the experiment. Feed ingredient samples were dried in a forced-air oven at 60°C for at least 24 hours, or until a constant weight was achieved, to determine the dry matter (DM) content [method no. 935.29 (AOAC 2012)]. Proximate analysis of ingredients was conducted using samples composited by month; N [method no. 968.06 (AOAC 2016); Rapid Max N Exceed, Elementar, Mt. Laurel, NJ], and ash [method no. 942.05 (AOAC 2012)]. Ether extract (EE) content analysis was conducted for MDGS and dried distillers grains plus solubles (DDGS) using a 5 L Soxhlet extractor (Thermo Fischer Scientific, Rockville, MD); tabular EE values were used for the remainder of the ingredients (NASEM 2016). Acid detergent fiber (ADF) and neutral detergent fiber (NDF) percentages were estimated to be 3% and 9%, respectively, for dry-rolled corn and high-moisture corn; fiber content analysis for all other ingredients was conducted as described by Goering and Van Soest (1970) with addition of sodium sulfite and alpha amylase. Actual diet formulation was based upon weekly DM determination and feed batching record with tabular energy values (Preston 2016) and are presented in Tables 1 and 2. Throughout the entire study, steers were offered 50% of their prescribed feed call twice daily and bunks were managed according to a slick-bunk management system (Pritchard 1993). However, during the first 97 d, bunks were managed to allow crumbs at the morning bunk read to ensure true ad libitum intake. Bunks were initially evaluated at 0730 each morning and this feed call was used to adjust daily as-fed feed allotment. A second evaluation of bunks was conducted and was targeted to be within 30 min prior to morning feeding for residual feed and a bunk score of 0 (slick) was targeted for all pens. The second daily bunk call was used to assist with determining the feed call for the following day after the initial bunk call to allow a more accurate bunk evaluation as there was > 2 h between the initial 0730 bunk call and actual feeding time. Determination of increasing as-fed feed allotment were evaluated by consecutive days of slick/crumbs (2 to 3 d) at the second bunk call and cattle behavior (ie aggressiveness in approaching the bunk at feeding time, time to clean up the morning feed call, distribution of cattle within the pen lying down, at the bunk, and standing, etc.) and feed call increases were made at 0.34 kg of DM/hd. Feed was manufactured twice daily in a single batch for each treatment in a stationary mixer (2.35 m^3^; Roto-Mix, Dodge City, KS), with a scale sensitivity of ± 0.45 kg. Feed was unloaded into a feed delivery wagon using the decreasing scale reading from the stationary mixer to allocate the correct amount of feed for each pen. Batching sequence was: (REC28), (REC56), and (REC84). Following each batch of feed, long-stem grass hay (∼1.8 kg) was added to the mixer and used to flush out all residual feed remaining in the mixer and re-used for each batch.

For determination of diet particle size and estimated physically effective NDF (peNDF), a weekly diet sample was obtained by sampling one replicate pen of each treatment and continuing weekly until all treatment replicate pens were sampled. This rotation continued until steers were no longer on differing diets (d 91). Quantification of particle size and peNDF was conducted using the Penn-State Particle Separator (PSPS) by multiplying the NDF content of the diet by the proportion of the diet that was > 4.0-mm (Kononoff et al. 2003).

Following the post-metaphylactic interval of the initial Draxxin KP administration, steers were pulse-dosed with chlortetracycline crumbles at 22.22 mg/kg of BW beginning on d 11 and this continued for 5 days. On d 97, steers were implanted with a 100 mg trenbolone acetate and 14 mg estradiol benzoate (Synovex-Choice; Zoetis Animal Health) implant and were re-implanted 92 d later (d 189) with a coated 200 mg trenbolone acetate and 28 mg estradiol benzoate (Synovex-ONE Feedlot; Zoetis Animal Health) implant. An implant retention check was conducted on d 126 and 217 by a trained technician. No severe implant site anomalies were noted on either day. Lubabegron fumarate (Experior; Elanco Animal Health) at 2.95 mg/kg of diet DM (36 mg/steer) was provided daily for 42 d and was withdrawn 4 d prior to slaughter.

### Blood collection

Following initial processing, the 2 steers closest to the mean pen BW on d 0 were selected for blood collection to quantify measures for serum urea-N (SUN), non-esterified fatty acids (NEFA), glucose, and cytokines. Whole blood samples were collected from sentinel steers (*n* = 2 steers/pen) into 10 mL non-additive tubes during the interim weighing process prior to the morning feeding on days 28, 42, 56, 70, 84, and 97 (relative to study initiation). Once collected, whole blood was transported to the Ruminant Nutrition Laboratory and centrifuged at 3100 × g at 4°C to extract sera within 2 hours following collection. Serum samples were stored at −80°C until subsequent laboratory analyses were completed.

Serum samples were analyzed for non-esterified fatty acids (NEFA) using a FujiFilm LabAssay NEFA kit (Wako Shibayagi Corporation, Chuo-ku, Osaka, Japan). Serum quantification of NEFA were measured using 4 µL determinations via colorimetric assay. The standard curve constructed for the NEFA assay was between 0 and 1.0 mEq/L. Serum samples were analyzed for urea-nitrogen (SUN) following the procedures of Fawcett and Scott (1960). Serum quantification of SUN was measured using 20 µL determinations via colorimetric assay. The standard curve constructed for the SUN assay was between 0 and 50 mg/dL. Serum samples were analyzed for glucose using a FujiFilm AutoKit Glucose kit (Wako Shibayagi Corporation, Cho-ku, Osaka, Japan). Serum quantification of glucose was measured using 2 µL determinations via colorimetric assay. The standard curve constructed for the glucose assay was between 0 and 200 mg/dL. Serum samples for all assays were analyzed in triplicate, and samples were considered for reruns if the CV among the absorbance values within triplicate determination was greater than 5%. The inter- and intra-assay CV was less than 15% for all assays. The R^2^ of the standard curve for all assays was *R*^2 = ^0.99.

### Health management

All steers removed from their home pen for health evaluation were then monitored in individual hospital pens prior to being returned to their home pens. When a steer was moved to a hospital pen, the steers daily allotment of feed (pen intake/number of steers per pen) from the home pen was removed and transferred to the hospital pen. If the steer in the hospital returned to their home pen, hospital pen feed was credited to the home pen. If the steer did not return to their home pen, all feed that was delivered to the hospital pen was deducted from the feed intake record for its home pen back to the date the steer was hospitalized. Health outcomes were characterized as: musculoskeletal (lameness), gastrointestinal (bloat), respiratory (pneumonia), other (pinkeye, etc.), removals, and general (dead). One steer was removed from the REC28 treatment group because of chronic bloat upon arrival to the study site and failure to improve after consecutive interventions. Two steers from the REC56 treatment were removed from the study on d 241 due to a non-ambulatory musculoskeletal injury. Cumulative mortalities included: two steers (REC28 and REC56), concluded to be caused by metabolic disorder (bloat) via necropsy. All events were determined to not be related to dietary treatment.

### Growth performance calculations

All steers were weighed individually at initial processing. Each following weighing event was targeted to occur every 28 days. However, to align with significant study events, weighing occurred on d 28, 56, 84, 97 (end of receiving period; implantation), 126, 168, 189 (terminal implant), 217 (implant retention check), 237 (beta-ligand feeding period), and 284 (shipment for slaughter). All BW measurements were obtained prior to the morning feeding. Cumulative growth performance was based upon initial BW (average BW at initial processing with a 4% shrink applied to account for digestive tract fill) and carcass-adjusted final BW, which was calculated as: (HCW/0.6285), where 0.6285 was the common study dressing percentage (DP%). Average daily gain (ADG) was calculated as the difference between final carcass-adjusted BW and initial shrunk BW, divided by days on feed; feed efficiency (G:F) was calculated from ADG/DMI.

### Carcass trait determination

On the morning of d 284, prior to morning feeding, a final BW was obtained, and electronic identification tags were administered for tag transfer at slaughter. Steers were offered 30% of the previous day’s feed allotment to minimize gut fill in anticipation of slaughter. Steers were shipped in the afternoon on d 284 and slaughtered the next day at a commercial slaughter abattoir. Visual and electronic identification were recorded in slaughter order to capture carcass data on an individual-animal basis. Liver abscess prevalence and severity were determined following evisceration according to the scoring system of Brown and Lawrence (2010): Edible (no abscesses), A− (1 or 2 small abscesses or inactive scars), A (1 to 2 large abscesses or multiple small abscesses), A+ (multiple large abscesses), A + Adhesion (liver adhered to gastrointestinal tract or diaphragm or both), or A + Open (open liver abscess). Hot carcass weight (HCW) was recorded during the slaughter procedure. Video image data were obtained from the plant for ribeye area (REA), rib fat thickness, and USDA marbling score. Assigned USDA yield grade was obtained from plant records. Estimated empty body fat (EBF) percentage and adjusted final BW at 28% EBF (AFBW) were calculated from observed carcass traits (Guiroy et al. 2002).

### Efficiency of dietary net energy utilization calculations

Observed dietary net energy (NE) and the ratio of observed-to-expected dietary NE were assessed to determine the efficiency of dietary net energy utilization during the first 97 d and the cumulative feeding period based on carcass-adjusted growth performance. Observed dietary NE was calculated from daily energy gain (EG; Mcal/d): EG = ADG^1.097^ × 0.0557 W^0.75^, where W is the mean equivalent BW [average of the initial shrunk BW and carcass-adjusted final BW × (478/AFBW), kg; (NASEM 2016)]. Maintenance energy required (EM; Mcal/d) was calculated by the following equation: EM = 0.077BW^0.75^ (Lofgreen and Garrett 1968) where BW is the mean feeding period BW (average of initial shrunk BW and carcass-adjusted final BW). However, the maintenance coefficient was increased to 0.08 to reflect the assumed lesser dietary energy utilization and increased maintenance energy requirements of Angus × Holstein steers compared with native beef steers (Carvalho et al. 2024). This relies on the assumption Angus × Holstein crossbred steers would be midway between the maintenance coefficient used for native beef and Holstein steers (0.077 vs 0.084; NASEM 2016). Using the estimates required for maintenance and gain, the observed dietary NE_m_ and NE_g_ values (Zinn et al. 2008) of the diet were generated using the quadratic formula: *x* = (−*b*±√(*b*^2^−4*ac*))/2*c*, where *x* = NE_m_, Mcal/kg, *a = *−0.41EM, *b = *0.877EM + 0.41DMI + EG, *c = *−0.877DMI, and NE_g_ was determined from: 0.877NE_m—_0.41 (Zinn and Shen 1998; Zinn et al. 2008). The maintenance coefficient (MQ) was determined from DMI, feed required for observed gain, and NE for maintenance of the diet using the following equation: ((DMI—feed required for gain) × (dietary NE_m_))/BW^0.75^, where feed required for gain was calculated from EG divided by dietary NE_g_. The ratio of observed-to-expected NE ratio was determined from observed dietary NE for maintenance or gain divided by tabular NE for maintenance or gain, a greater ratio than 1.0 indicates better than expected utilization of dietary net energy based upon tabular estimates for dietary net energy and DMI.

### Statistical analysis

Growth performance, carcass traits, and efficiency of dietary NE utilization were analyzed using the GLIMMIX procedure of SAS 9.4 (SAS Inst. Inc., Cary, NC) in a randomized complete block design. For growth performance, carcass traits, and efficiency of dietary NE utilization analyses, the effect of dietary treatment was tested with a random effect of pen replicate. Pen served as the experimental unit for all analyses. Least squares means were generated using the LSMEANS statement of SAS, and treatment effects were analyzed using the pairwise comparisons PDIFF and LINES option of SAS 9.4. Distributions of USDA quality grade (QG) and yield grade (YG), as well as liver abscess prevalence and severity, were analyzed as binomial distributions using the GLIMMIX procedure of SAS 9.4 to quantify differences in the distributions among treatments. Hourly and daily rumination behavior data were analyzed using the MIXED procedure of SAS. Daily rumination behavior was analyzed with dietary treatment, days on feed (DOF), and the treatment × DOF interaction as fixed effects in the model. For hourly rumination behavior, the model included the fixed effects of dietary treatment, hour, and the treatment × hour interaction. For both analyses, DOF and hour were considered repeated measures, and autoregressive was selected as the covariance structure based on the lowest Akaike Information Criterion (AIC) value. Serum-NEFA, SUN, and glucose were averaged by pen and analyzed using the same random and fixed effects as described previously. Serum outcomes were analyzed as repeated measures with d 28 used as a covariate in the model as all treatments remained on the same diet until d 28. The covariance structure selected was autoregressive for all serum analyses based on the lowest AIC value. Least squares means were generated using the LSMEANS statement of SAS. For all analyses, an *α* of 0.10 or less was used to determine significance, and tendencies are discussed between 0.10 and 0.15.

## Results and discussion

### Receiving period growth performance

Receiving period (d 1 to 97) growth performance is reported in Table 3. As expected, no differences in growth performance outcomes (*P* ≥ 0.38) were observed during the initial 28 d as steers remained on the same diet (30% RE) until REC28 steers transitioned to the 21R diet beginning on d 28 following study initiation. Consuming the receiving diet greater in forage concentration compared to the finishing diet appeared to limit DMI, as the REC56 and REC84 steers consumed less DM (*P* < 0.01) compared to REC28 steers from d 29 to d 56 and d 1 to 56, respectively. From d 57 to 84, remaining on the receiving ration tended to negatively influence DMI (*P* = 0.15), with the REC84 steers consuming numerically less DM than REC28 and REC56 steers, respectively. By d 84, DMI was not different (*P* = 0.37) between REC28 and REC56 steers after REC56 had spent 28 days on a finishing ration; however, REC84 steers had consumed 3.9% less DM than REC28 steers (*P* = 0.02), but did not differ from REC56 steers (*P* = 0.31). Throughout the 97-d receiving period, a reduction in DMI (*P* = 0.10) was observed with increasing time spent on a high-forage receiving diet, where REC28 steers consumed the most DM, differing from both REC56 and REC84 steers (*P* ≤ 0.10), with the REC56 not differing from REC84 steers (*P* = 0.65). Likewise, Pritchard and Mendez (1990) observed calves fed high-concentrate diets consumed more DM than calves fed a corn silage-based diet for the first 28 and 56 d in the feedlot. Prior to feedlot arrival, calves in the present study were consuming 3% to 4% of their BW in DM of a texturized calf starter containing 43.6% starch and not offered any additional roughage. Therefore, it is likely that transitioning dairy-derived calves from a texturized calf starter to a feedlot receiving ration, particularly one that contains increased amounts of a low-DM, bulky, ensiled roughage source such as corn silage, may reduce intake due to gut fill limitations. As DMI is a driver of energy intake and thus body weight gain, it is not surprising a detrimental effect on ADG and BW were observed with additional days fed the receiving diet. Greater length of time spent on the receiving diet reduced ADG from d 29 to 56 and d 1 to d 56, d57 to 84, and d 1 to 84 (*P* ≤ 0.09). By d 97, a 10% advantage in ADG was observed in REC28 steers compared to REC84 steers (*P* < 0.01), thus resulting in the REC28 steers weighing 15 kg heavier than REC84 by d 97 (*P* < 0.01). Steers on the REC84 treatment consumed less DM (*P* = 0.10) during the initial 97 DOF, thus promoting a negative response (*P* < 0.01) on G:F (0.194 vs. 0.209) compared to the REC28 and REC56 steers. However, G:F was similar at all other interim time points (*P* ≥ 0.34), suggesting that the observed reduction in ADG was because of an equivalent response to reduced DMI and energy intake. As expected, increased time spent on a diet with greater energy-density resulted in increased performance-based NE for maintenance and gain (*P* < 0.01). Calculated maintenance coefficient (MQ) increased with less time spent on the receiving diet (*P* = 0.04). Whereas REC28 steers had a greater maintenance coefficient compared to REC56 and REC84 steers (*P* ≤ 0.08; 0.111 vs 0.108 and 0.106, respectively), with REC56 and REC84 steers not differing (*P* = 0.35). The maintenance coefficient for Holstein steers is adjusted to 0.084 from 0.077 in beef steers (NASEM 2016). Therefore, it is assumed that beef × dairy steers would be intermediate (0.080 to 0.082; Carvalho et al. 2024). Carvalho et al. (2024) confirmed the enhancement of dietary NE utilization in Angus × Holstein steers compared to Holstein steers via a reduction in maintenance energy expenditures. However, the MQ values generated in the current study during the first 97 d were between 25 and 28% greater than the midway value for beef × dairy steers proposed by Carvalho et al. (2024). However, the MQ reported by Carvalho et al. (2024) was calculated based on entire-feeding period performance, whereas values from the present study were calculated based on the first 97 d. These deviations from expected performance may be partly explained by the rapid transition to a less energy-dense, forage-based diet compared to the previously consumed, energy-dense calf starter. Therefore, the steers in the present study may have been attempting to eat to an equivalent energy intake to the previous diet, which may explain the 14 to 17% increase in observed-to-expected DMI across all treatments. The utilization of energy by cattle can vary depending on their physiological status (ie growing or mature; Hales 2019). Furthermore, derived relationships between NE intake and RE and rate of gain from NRC and NASEM equations may not apply to cattle fed diets providing increased roughage (Zinn et al. 2008). Therefore, because of the rapid diet change, steers in the current study may have had less efficient use of energy intake via alterations to rumen fermentation capacity, thus increasing their maintenance coefficient beyond what was anticipated. However, this could be indicative of changes in maintenance energy requirements with changes in gut mass via increasing forage intake. McLeod and Baldwin (2000) observed increased visceral organ mass in lambs fed 75% forage compared to lambs fed 75% concentrate, concluding that increased forage intake increases gut energy expenditure. In the present study, the observed-to-expected ratio of NE for maintenance tended to differ among treatments (*P* = 0.12) but did not differ for NE for gain (*P* = 0.34). However, the observed-to-expected ratio of DMI and ADG for maintenance and gain was not different (*P* ≥ 0.19) among treatments. Regardless of treatment, based on energetic calculations from known dietary energy intake and observed performance, steers consumed 14% to 17% more DM, gained 19% to 20% less, and had 10% to 16% worse utilization of NE for maintenance and gain than expected during the initial 97 d.

**Table 3:** Receiving period performance (d 1–97) of Angus × Holstein steers fed a receiving diet for 28 (REC28), 56 (REC56), or 84-d (REC84).

	Treatment				*P*-value
Item^1^	REC28	REC56	REC84	SEM	*F*-test
**Pens, *n***	6	6	6	–	–
**Initial BW, kg**	192	192	192	0.34	0.94
**BW, kg**					
** d 28**	228	227	228	1.0	0.61
** d 56**	276^a^	271^b^	272^b^	1.9	0.02
** d 84**	322	320	315	2.7	0.12
** d 97**	340^a^	331^b^	325^b^	2.8	<0.01
**ADG, kg**					
** d 1–28**	1.29	1.25	1.28	0.033	0.38
** d 29–56**	1.71^a^	1.56^b^	1.56^b^	0.054	0.02
** d 57–84**	1.65^ab^	1.75^a^	1.57^b^	0.080	0.08
** d 1–56**	1.50^a^	1.41^b^	1.42^b^	0.032	<0.01
** d 1–84**	1.55^a^	1.52^ab^	1.47^b^	0.032	0.09
** d 1–97**	1.52^a^	1.43^b^	1.37^b^	0.029	<0.01
**DMI, kg**					
** d 1–28**	4.87	4.84	4.90	0.036	0.50
** d 29–56**	7.69^a^	7.03^b^	7.03^b^	0.115	<0.01
** d 57–84**	8.72	8.88	8.50	0.131	0.15
** d 1–56**	6.28^a^	5.93^b^	5.96^b^	0.069	<0.01
** d 1–84**	7.09^a^	6.92^ab^	6.81^b^	0.071	0.05
** d 1–97**	7.30^a^	7.11^b^	7.05^b^	0.087	0.10
**G:F**					
** d 1–28**	0.263	0.258	0.263	0.0059	0.59
** d 29–56**	0.223	0.222	0.221	0.0074	0.97
** d 57–84**	0.189	0.196	0.184	0.0080	0.34
** d 1–56**	0.239	0.237	0.238	0.0056	0.90
** d 1–84**	0.219	0.220	0.215	0.0041	0.61
** d 1–97**	0.209^a^	0.201^b^	0.194^c^	0.0025	<0.01
**Cumulative energetics** ^2^					
** Obs. NE_m_**	1.76^a^	1.73^a^	1.68^b^	0.015	<0.01
** Obs. NE_g_**	1.13^a^	1.10^a^	1.07^b^	0.013	<0.01
** MQ**	0.111^a^	0.108^b^	0.106^b^	0.0019	0.04
** O:E DMI**	1.17	1.15	1.14	0.012	0.19
** O:E ADG**	0.80	0.81	0.81	0.013	0.45
** O:E NE_m_**	0.88	0.89	0.90	0.008	0.12
** O:E NE_g_**	0.84	0.85	0.86	0.010	0.34

a,b,c Means within a row lacking a common superscript differ *P* ≤ 0.05.

1 All BW measures were pencil shrunk 4% to account for digestive tract fill.

2 MQ: Calc. maintenance coefficient: (((DMI—feed required for gain) × (dietary NE_m_))/W^^0.75^); O:E: ratio of observed to expected performance.

### Finishing period growth performance and carcass characteristics

Post-receiving period and cumulative growth performance are reported in Table 4. Post-receiving period ADG, DMI, and G:F were similar (*P* ≥ 0.18) regardless of receiving period management. When DMI was evaluated throughout the feeding period (Fig. 1), there was no treatment × day effect (*P* = 0.65) or treatment effect (*P* = 1.00), but an effect of day (*P* < 0.01) was observed. As expected, DMI increased with increasing days on feed. Despite similar DMI after the first 97 d, steers spending a greater amount of time on the receiving diet were never able to recapture the loss of BW gain realized during the initial 97 d. Live BW at d 168 was greater (*P* = 0.10) with less time spent on the receiving diet. After 77 d on a common finishing diet, the difference in BW (11 kg) between REC28 and REC84 calves was still apparent. The disparity in BW was still observed throughout the remainder of the feeding period, reflected in the numerically greater carcass-adjusted final BW (*P* = 0.32) with less time spent on the receiving diet, resulting in REC84 steers weighing 16 kg less than REC28 steers on a carcass-adjusted basis. Cumulatively, numerically greater ADG and G:F was observed with less time spent on the receiving ration, but entire feeding-period DMI did not differ (*P* = 1.00) among treatments. Although REC84 steers were able to consume a similar amount of DM compared to REC28 and REC56 steers for the remainder of the feeding period, the numerical disparity in BW was never recovered. When BW was adjusted to 28% EBF (AFBW), no differences (*P* = 0.84) in AFBW among treatments were observed. Furthermore, no differences (*P* ≥ 0.18) in performance-based NE for maintenance and gain, MQ, or observed-to-expected ratios of DMI, ADG, NE_m_, and NE_g_ were observed. Calculated MQ values in the current experiment were slightly greater (0.084) compared to reported values by Carvalho et al. (2024) but still greater compared to native beef cattle (0.077; NASEM 2016). This confirms the greater maintenance requirements of dairy-influenced steers compared to beef steers.

**Figure 1:**
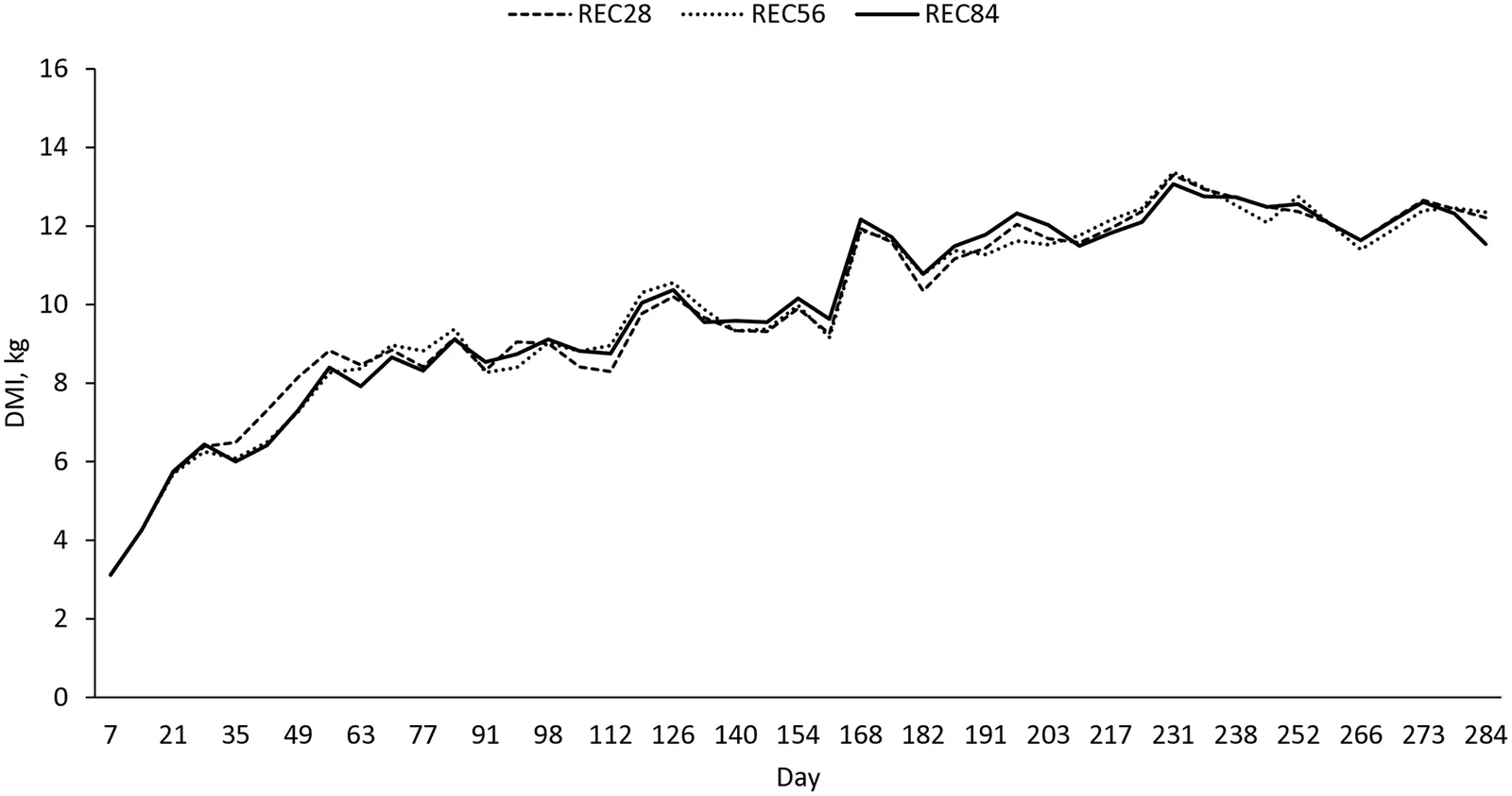
Daily dry matter intake, kg (DMI) of Angus × Holstein steers fed a receiving diet for either 28 (REC28), 56 (REC56) or 84-d (REC84) post feedlot-arrival through d 284. Effect of treatment, *P* = 1.00; Effect of day, *P* < 0.01; Treatment × day, *P* = 0.65. Standard error of the mean = 0.2965 kg/d.

**Table 4:** Post-receiving phase and cumulative growth performance of Angus × Holstein steers fed a receiving diet for 28 (REC28), 56 (REC56) or 84-d (REC84).

	Treatment				*P*-value
Item	REC28	REC56	REC84	SEM	*F*-test
**Pens, *n***	6	6	6	–	–
**d 168 BW, kg** ^1^	479^a^	475^ab^	468^b^	3.2	0.10
**Carcass-adjusted Final BW, kg** ^2^	680	677	664	7.8	0.32
**AFBW, kg** ^3^	607	602	604	5.4	0.84
**ADG, kg**					
** d 97–168**	1.96	2.03	2.02	0.027	0.20
** d 97–284** ^2^	1.82	1.85	1.81	0.043	0.74
** d 168–284** ^2^	1.73	1.75	1.69	0.059	0.70
** d 0–284** ^2^	1.72	1.71	1.66	0.027	0.32
**DMI, kg**					
** d 97–168**	9.60	9.82	9.85	0.110	0.18
** d 97–284**	11.09	11.19	11.20	0.210	0.85
** d 168–284**	12.01	12.03	12.03	0.287	1.00
** d 0–284**	9.80	9.79	9.78	0.156	1.00
**G:F**					
** d 97–168**	0.205	0.207	0.205	0.0033	0.87
** d 97–284** ^2^	0.164	0.166	0.162	0.0034	0.70
** d 168–284** ^2^	0.145	0.145	0.140	0.0041	0.59
** d 0–284** ^2^	0.176	0.175	0.170	0.0025	0.26
**Cumulative (d 0 to 284)** ^4^					
** Obs. NE_m_**	2.00	2.00	1.95	0.022	0.18
** Obs. NE_g_**	1.34	1.34	1.30	0.019	0.18
** MQ**	0.084	0.082	0.086	0.0027	0.62
** O:E DMI**	1.02	1.01	1.03	0.013	0.62
** O:E ADG**	0.97	0.98	0.96	0.019	0.66
** O:E NE_m_**	0.98	0.99	0.98	0.011	0.66
** O:E NE_g_**	0.98	0.99	0.97	0.014	0.64

1 All BW measures were pencil shrunk 4% to account for digestive tract fill.

2 Adjusted final BW to a common study DP of 0.6285; Carcass adjusted FBW = (HCW/0.6285).

3 Final BW adjusted to 28% EBF (Guiroy et al. 2002).

4 MQ = Calc. maintenance coefficient: (((DMI—feed required for gain) × (dietary NE_m_))/W^^0.75^); O:E: ratio of observed to expected performance.

Carcass outcomes are reported in Table 5. Dietary treatment affected 12th rib fat thickness (*P* = 0.07), with steers on the REC84 treatment being leaner at slaughter compared with REC28 and REC56 (*P* ≤ 0.04; 1.36 vs. 1.53 cm, respectively), with REC28 and REC56 not differing (*P* = 1.00). No other differences were observed (*P* ≥ 0.19) in any other carcass outcomes measured. Although not statistically different (*P* = 0.31), steers on the REC28 and REC56 treatment exhibited a numerical advantage in HCW compared to REC84 steers (427 and 426 vs 417 kg, respectively). Categorical carcass outcomes are reported in Table 6. The distributions of quality and yield grade were not altered (*P* ≥ 0.40) by receiving period management. However, steers on the REC84 treatment resulted in a numerically greater proportion of Yield Grade 2 and a lesser proportion of Yield Grade 4 carcasses as compared to REC56 and REC28 steers. No difference in the prevalence of total liver abscesses (*P* = 0.78) or prevalence of major abscesses (*P* = 0.62) was observed among treatments. However, a numerical reduction in total abscess prevalence was noted with greater time spent on the receiving diet (REC28 & REC84; 41.3 vs. 33.3%), driven by a tendency (*P* = 0.13) for a decrease in the prevalence of minor abscesses due to dietary treatment. Dietary inclusion of roughage has been reported to influence the prevalence of liver abscesses in cattle at slaughter (Zinn and Plascencia 1996; Word et al. 2024). To determine the effects of timing of roughage inclusion, Loerch and Fluharty (1998) evaluated concentrate level in the diet (85 and 100%) during the growing (initial 84 d) and finishing phase of beef steers fed for 186 d. From the study, growing phase concentrate level had little influence on the prevalence of condemned livers. Furthermore, regardless of growing phase concentrate level, steers fed 100% concentrate for the finishing period resulted in twice the prevalence of condemned livers compared to 85% concentrate during the finishing period. Effects of receiving-period diet formulation on prevalence of liver abscesses are difficult to accomplish because of the extended time between the receiving period and slaughter endpoint (Bryant and Jennings 2022), as observed in the present experiment. While a promising strategy to help mitigate the occurrence of liver abscesses at slaughter in beef × dairy cattle, the effect of increasing time spent on the receiving ration on both receiving period- and entire-feeding period growth and carcass performance may not be economical for all feeding systems. Dairy-influenced cattle are associated with having greater prevalence of liver abscesses at slaughter (Amachawadi and Nagaraja 2016). This is likely because of the increased time spent on feed, particularly on high-concentrate finishing diets, compared to native beef cattle. Furthermore, dairy-influenced cattle have greater maintenance requirements as compared to native beef cattle (NASEM 2016), thus requiring more feed for maintenance. This allows for more opportunities for them to encounter periods of acidosis and bloat, and a longer period spent under acidotic conditions, making them more susceptible to liver abscesses.

**Table 5:** Carcass traits of Angus × Holstein steers fed a receiving diet for 28 (REC28), 56 (REC56), or 84-d (REC84).

	Treatment				*P*-value
Item	REC28	REC56	REC84	SEM	*F*-test
**Carcasses, *n***	46	45	48	–	–
**HCW^1^, kg**	427	426	417	4.9	0.31
**DP^2^, %**	62.97	62.84	62.72	0.294	0.80
**REA^3^, cm^2^**	89.74	88.69	88.07	1.078	0.55
**Rib fat, cm**	1.53^a^	1.53^a^	1.36^b^	0.055	0.07
**Calculated YG**	3.49	3.52	3.33	0.087	0.19
**Marbling score^4^**	520	525	516	15.9	0.91
**Estimated EBF^5^, %**	31.80	31.93	31.10	0.374	0.28

1 Hot carcass weight, kg (HCW).

2 Dressing percentage (DP, %) = (hot carcass weight/final body weight, shrunk 4%) × 100.

3 Ribeye area, cm^2^ (REA).

4 400 = Small^00^.

5 Estimated percentage empty body fat calculated from Guiroy et al. (2002) = 17.76207 + (4.68142 × fat thickness, cm) + (0.01945 × hot carcass weight, kg + (0.81855 × QG) – (0.06754 × ribeye area, cm^2^); QG: quality grade (Select = 4, Low Choice = 5, Average Choice = 6, High Choice = 7, Low Prime = 8).

**Table 6:** Categorical carcass outcomes of Angus × Holsteins steers fed a receiving diet for 28 (REC28), 56 (REC56), or 84-d (REC84).

	Treatment			*P*-value
Item	REC28	REC56	REC84	*F*-test
**Carcasses, *n***	46	45	48	–
**Quality grade, %**				
** Prime**	8.7	4.5	6.7	0.79
** Choice or better**	84.8	95.5	86.7	0.52
** Select**	15.2	4.5	13.3	0.52
**Yield grade, %**				
** 2**	17.4	18.2	25.5	0.54
** 3**	65.2	61.4	63.8	0.92
** 4**	17.4	20.4	10.7	0.40
**Liver outcomes^1^, %**				
** Edible**	58.7	62.2	66.7	0.78
** Abscessed**	41.3	37.8	33.3	0.78
** Minor**	23.8	17.4	8.2	0.13
** Major**	17.4	20.0	25.0	0.62

1 Evaluated according to the scoring system of Brown and Lawrence (2010): Edible (no abscesses); Minor = A− (1 or 2 small abscesses or inactive scars), A (1 to 2 large abscesses or multiple small abscesses); Major = A+ (multiple large abscesses), A + Adhesion (liver adhered to gastrointestinal tract or diaphragm or both), or A + Open (open liver abscess).

### Particle separation

Roughage inclusion can range from 8% to 10% and > 40% dietary DM in finishing and receiving cattle feedlot diets, respectively (Samuelson et al. 2016). However, the inclusion and dietary NDF contribution of roughages alone provide little information regarding the effectiveness of the roughage source in stimulating chewing and rumination activity. In finishing cattle diets, roughage is typically included as a functional ingredient, making little contribution to the energy density of the diet but providing necessary characteristics to maintain rumination, ruminal pH, and reduce risk of ruminal acidosis (Jennings et al. 2020). Hence, formulating diets for peNDF concentration can be used as a strategy to optimize roughage use without increasing roughage inclusion and maximizing animal performance (Fox and Tedeschi 2002). Particle separation results using the PSPS for each diet fed are reported in Table 7. As expected, the 30R diet contained the greatest (*P* < 0.01) proportion of particles retained on the 19- and 8-mm sieves and the least proportion of particles retained on the 4-mm sieve compared to the 21R and 12R diets. The 12R diet contained the greatest proportion of particles < 4-mm, but the least proportion of particles > 4-mm (*P* < 0.01). Hence, the estimated percentage peNDF offered by the 30R was the greatest among treatment diets, followed by the 21R and 12R diets (*P* < 0.01; 29.3, 22.4, and 17.0%, respectively). The recommended dietary peNDF value to maximize animal performance varies in published literature, with dietary peNDF concentrations ranging from 12 to 15% (Mertens 2002) and 7 to 10% (Fox and Tedeschi 2002). Regardless, the 12R finishing diet used in the present study was well in excess of the recommended values for peNDF.

**Table 7:** Particle separation and estimated peNDF of diets fed through d 91.

	Diets^1^				*P*-value
Item	30R	21R	12R	SEM	*F*-test
**No. of samples**	24	3	12	–	–
**NDF, % DM**	33.5	27.5	22.5	–	–
**Sieve size, mm**					
** 19.0**	12.0^a^	6.8^b^	2.8^c^	0.80	<0.01
** 8.0**	57.3^a^	48.8^b^	36.7^c^	1.25	<0.01
** 4.0**	18.5^c^	26.7^b^	36.1^a^	0.43	<0.01
** Particles < 4 mm**	12.2^c^	18.2^b^	24.5^a^	1.15	<0.01
** Particles > 4 mm**	87.8^a^	81.8^b^	75.5^c^	1.15	<0.01
**Estimated peNDF^2^, % of DM**	29.3^a^	22.4^b^	17.0^c^	0.55	<0.01

a,b,c Means within a row lacking a common superscript differ *P* ≤ 0.05.

1 30R: 30% roughage equivalent diet; 21R: 21% roughage equivalent diet; 12R: 12% roughage equivalent diet.

2 Percent peNDF was estimated by multiplying the percentage of sample > 4-mm in particle size by the percent NDF of the diet.

### Rumination behavior

Daily rumination activity in min/d is presented in Fig. 2. A treatment × day interaction was observed (*P* < 0.01) for minutes spent ruminating/d. Treatment differences in rumination behavior were observed at d 8, 17, and 22 (*P* ≤ 0.09), which was unexpected as dietary treatments were not initiated until d 28. Therefore, this response is difficult to explain. Differences in rumination behavior were not observed again until d 30 (*P* = 0.10), 2 d following the REC28 steers transition to the 21R diet. Across the 97-d receiving period, rumination activity only differed on d 30, 31, 36 to 38, 45, 48, 50 to 56, 58 to 60, 64, 66, 70 to 72, 76, 78, 80, and 94. Crawford et al. (2022) fed either a lower energy, higher roughage receiving diet or a high-energy finishing diet to newly received beef calves during the first 56 d. Cattle consuming the receiving diet spent more time ruminating on days 1 to 14, 17 to 24, 26, and 28 of the study. However, the authors observed no differences in rumination activity between d 28 and 56, despite differences in roughage inclusion between the two treatments. This response was also observed in the current study (Fig. 2). Greater length of time spent on the receiving diet resulting in increased (*P* < 0.01) daily rumination activity during the 97 d receiving period. Whereas REC28 steers ruminated the least (min/d) followed by REC56, and REC84 ruminated the most (*P* < 0.01; 518, 523, and 532 min/d. respectively). This response was expected as steers on the REC84 treatment consumed the 30R diet the longest and thus consumed more peNDF cumulatively. Physically effective NDF is described as the portion of the NDF that contributes to the roughage’s ability to stimulate rumination (Mertens 1997), with increasing peNDF resulting in increased daily rumination (Beauchemin and Yang 2005). Regardless of treatment, steers in the present study exhibited increased rumination activity (524 min/d) compared to other published values in native beef steers fed a feedlot diet (250 to 350 min/d; Gentry et al. 2016; Pillmore et al. 2024). However, the values observed in the present study more closely match daily rumination values reported in dairy cows (400 to 500 min/d; Schirmann et al. 2016; Ben Meir et al. 2018). Carvalho et al. (2020) quantified differences in rumination behavior among Angus and Holstein steers fed either grain or forage-based diets. The authors observed increased rumination activity in Holstein steers compared to Angus steers in both the grain (468 vs. 382 min/d) and forage-based (577 vs. 502 min/d) diets. Still, the reported values for the aforementioned study in Holstein steers fed a grain-based diet are less than the values observed in the current study. The steers in the present study were much lighter than the Holstein steers used by Carvalho et al. (2020; 192 vs. 483 kg). However, steers in the present study consumed a much greater percentage of BW in DM compared to the Holstein steers used by Carvalho et al. (2020; 2.72 vs. 2.13%). Bae et al. (1983) evaluated the relationship between body size and rumination activity of dairy cows and steers and reported decreased rumination time (500 to 455 min/d) with increasing body weight from 261 to 891 kg. This supports the observed decrease (*P* < 0.01) in rumination activity as both days on feed and body weight increased over the 97-d receiving period across all treatments. When considering animal age and chewing capacity, Gorocica-Buenfil and Loerch (2005) observed no effect of calf age (weanling vs yearling) on chewing capacity of diets consisting of whole shelled corn or ground corn as evidenced by the similar fecal starch concentration between calf ages.

**Figure 2:**
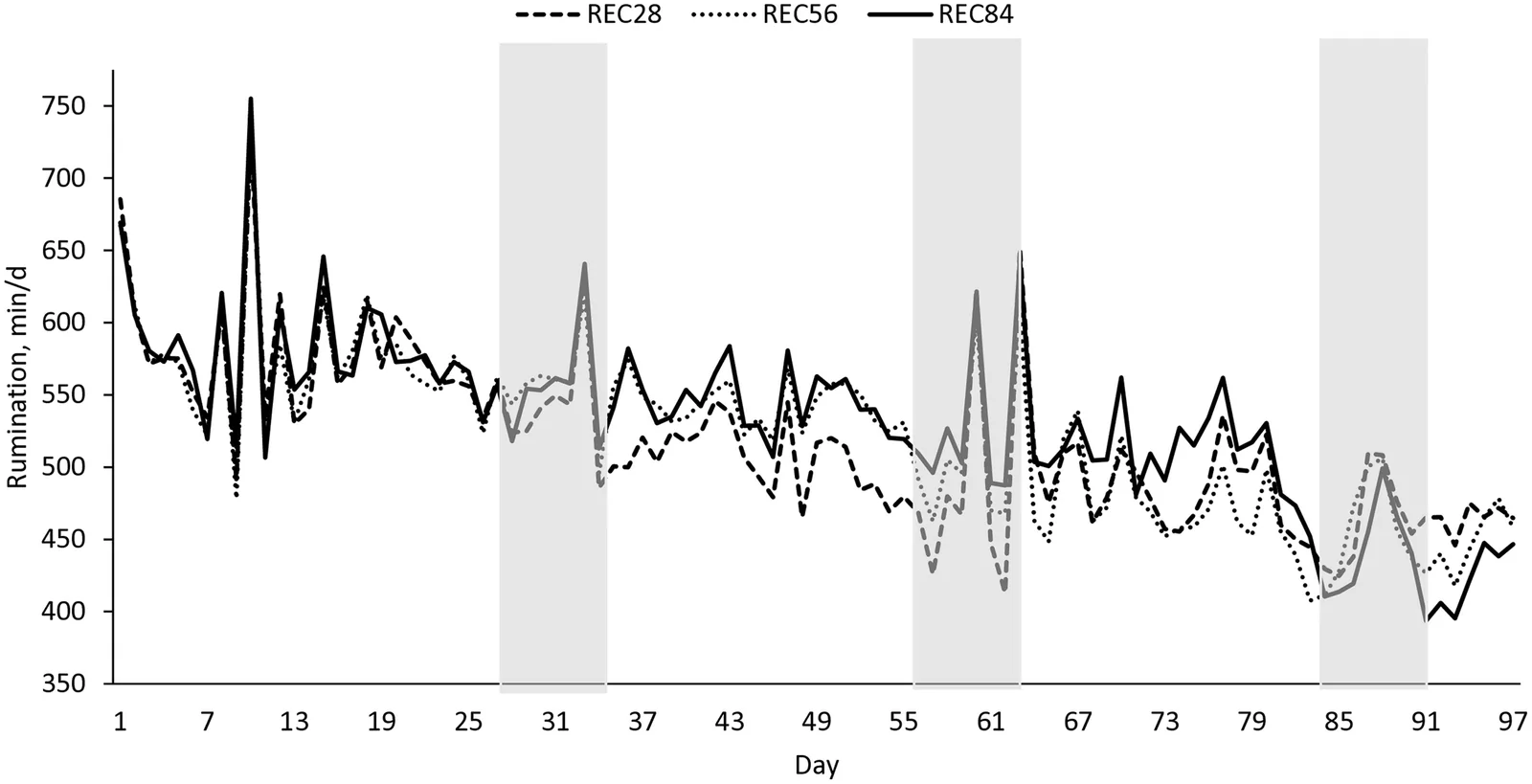
Daily rumination (min/d) of Angus × Holstein steers fed a receiving diet for either 28 (28REC), 56 (REC56) or 84 d (REC84) post feedlot-arrival through d 97. All steers received a 3-axis accelerometer tag (Allflex Livestock Intelligence, Madison, WI) to quantify daily rumination activity. Shaded areas indicate when steers began the intermediate (21R) diet for 7 d prior to initiation of the 12R finishing diet as according to treatment. Effect of treatment, *P* < 0.01; day, *P* < 0.01; treatment × day, *P* < 0.01. Standard error of the mean = 14.85 min/d.

Hourly rumination activity is reported in Fig. 3. Steers were fed twice daily at 1000 and 1600 h. A treatment × hour interaction was observed (*P* < 0.01) for minutes spent rumination/h. At 0200 h, rumination activity differed (*P* = 0.06) between REC56 and REC84 steers (31.02 vs. 31.98 min/h), but were both greater (*P* < 0.01) compared to REC28 (29.56 min/d). Rumination peaked at 0400 h; and was not different (*P* = 0.20) between REC56 and REC84 steers (30.99 vs. 31.59 min/h), but were both greater (*P* ≤ 0.05) than REC28 steers (30.03 min/h). No differences (*P* ≥ 0.18) in hourly rumination activity among treatments were observed at 0600, 0800, or 1000 h. Although rumination activity was the least at 1000 h (9.02 min/h; *P* < 0.01) across all treatments. Interestingly, at 1200 h, steers on the REC28 ruminated the most (*P* = 0.02), but did not differ from REC56 steers (*P* = 0.20), with both REC28 and REC56 steers spending more time ruminating than REC84 steers (*P* ≤ 0.07). It is unclear why steers on the REC28 treatment spent more time ruminating from 1000 to 1200 h, whereas at nearly every other timepoint they spent the least time ruminating. Therefore, this response is difficult to explain. However, differences in diet composition, including greater inclusion of corn silage in the 30R diet, could have resulted in differences in eating behavior, thus influencing hourly rumination activity, though not measured in the present study. Greater inclusion of roughage and longer particles would require more chewing and/or rumination activity to reduce the size of roughage particles for passage from the rumen. Therefore, steers on the 12R diet could, potentially, finish a meal and return to a resting state of rumination before steers on the 30R diet. No differences (*P* ≥ 0.51) in hourly rumination activity among treatments were observed at 1400, 1600, 1800, or 2000 h. At 2200 and 0000 h, rumination differed between all treatments (*P* ≤ 0.06), with the REC84 ruminating the most followed by REC56 and REC28. Overall, dietary treatment affected hourly rumination time (*P* < 0.01), whereas REC84 steers spent the most time ruminating over the 97-d receiving period, followed by REC56, with steers on the REC28 treatment spending the least time ruminating on an hourly-basis. This was expected, as steers on the REC84 treatment spent the greatest time on the 30R diet and this is consistent with the findings of Weiss et al. (2017), whereas increased roughage inclusion resulted in increased time spent ruminating.

**Figure 3:**
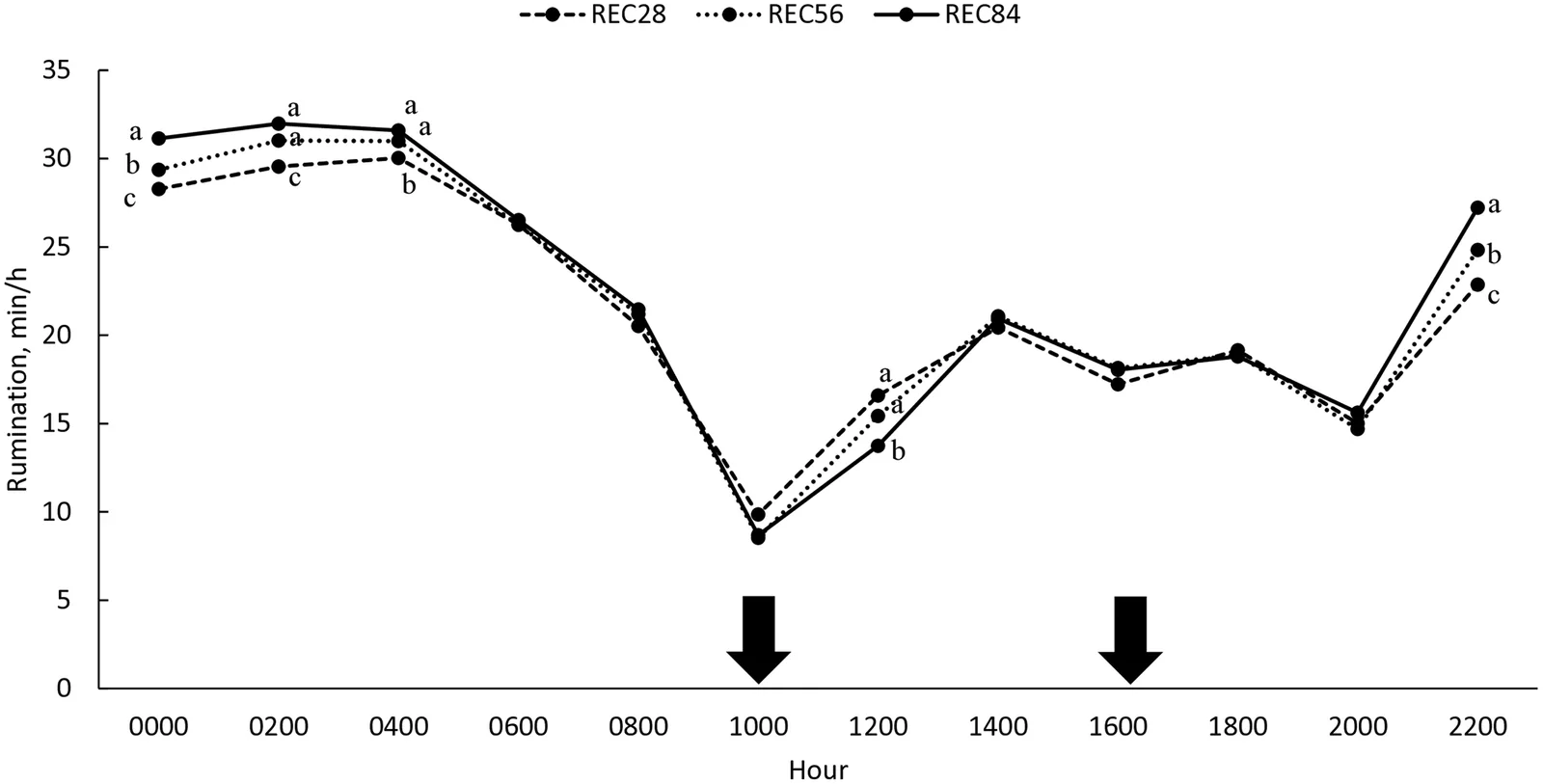
Circadian hourly rumination of Angus × Holstein steers fed a receiving diet for either 28 (REC28), 56 (REC56) or 84 d (REC84) post feedlot-arrival. All steers received a 3-axis accelerometer tag (Allflex Livestock Intelligence, Madison, WI) to quantify daily rumination activity. Arrows indicate time of feeding (1000 h & 1600 h, respectively). Effect of treatment, *P* < 0.01; hour, *P* < 0.01; treatment × hour, *P* < 0.01. Standard error of the mean = 0.5474 min/h. ^a-c^Within an hour, treatment means without a common superscript differ (*P* ≤ 0.10).

### Rumination and particle separation

Comparisons of rumination activity and diet characteristics as obtained from the PSPS were evaluated during the first 91 d rather than the first 97 d as steers were on differing diets until steers on the REC84 treatment transitioned to the 12R finishing ration on d 91 (Table 8). Dry matter intake was not different (*P* = 0.70) during the initial 91 d, whereas rumination time was different (*P* < 0.01) across day. Days spent on each diet (30R, 21R, and 12R) were weighted according to each dietary treatment (REC28, 56, or 84) to estimate kg of NDF and peNDF consumed/d based on DM consumed/d and the proportion of % NDF and peNDF offered by each diet. Average daily consumption of NDF and peNDF increased (*P* < 0.01) with increasing time spent on a high-forage receiving diet. Length of time spent on a high-forage receiving diet did not alter (*P* = 0.65) minutes spent ruminating per kg of DM. Differing from the present study, Gentry et al. (2016) and Tomczak et al. (2019) both reported increased rumination per kg of DM in cattle consuming diets with a greater inclusion of roughage. However, in the present study when evaluating kg of NDF and peNDF consumed per minute spent ruminating/d, differences among treatments (*P* ≤ 0.02) were observed. A decrease (*P* < 0.02) in minutes ruminating/kg of NDF consumed was observed with increased time spent on the receiving diet. Steers on the REC28 treatment spent the most time ruminating/kg of NDF consumed (*P* < 0.01; 304.4 min/kg), and REC84 steers spent the least amount of time ruminating/kg of NDF consumed (259.2 min/kg), with REC56 steers being intermediate (281.1 min/kg), but tending to differ (*P* = 0.13) from REC28 and REC84 steers. Similarly, a decrease (*P* < 0.01) in rumination minutes/kg of peNDF consumed was observed with increased time spent on a receiving ration. Steers on the REC28 treatment spent the most time ruminating/kg of peNDF consumed (*P* < 0.01; 381.0 min/kg), followed by REC56 steers (333.1 min/kg), with REC84 steers spending the least amount of time ruminating/kg of peNDF consumed (294.5 min/kg). Crawford et al. (2022) also observed increased rumination activity per kg of NDF intake in calves fed a finishing diet compared to a higher-roughage receiving diet, suggesting that factors other than diet may influence rumination activity. However, Gentry et al. (2016) observed no difference in rumination activity per kg of NDF and peNDF consumed with increased roughage inclusion (5% short-grind cornstalks vs 10% short-grind cornstalks).

**Table 8:** Rumination activity (d 1 to d 91^1^) of Angus × Holstein steers fed a receiving diet for 28 (REC28), 56 (REC56) or 84-d (REC84).

	Treatment				*P*-value
Item	REC28	REC56	REC84	SEM	*F*-test
**Rumination, min/d**	515.9^b^	527.7^ab^	540.8^a^	6.75	0.01
**DMI, kg/d**	7.19	7.02	6.94	0.210	0.70
**NDF intake, kg/d**	1.77^c^	2.00^b^	2.36^a^	0.058	0.01
**peNDF intake, kg/d**	1.39^c^	1.67^b^	2.04^a^	0.046	0.01
**Rumination, min/kg**					
** DM**	81.3	84.8	86.9	4.31	0.65
** NDF**	304.4^a^	281.1^ab^	259.2^b^	10.89	0.02
** peNDF**	381.0^a^	333.1^b^	294.5^c^	11.47	0.01

a,b,c Means within a row lacking a common superscript differ *P* ≤ 0.05.

1 d 91 was considered for comparisons of rumination activity and diet characteristics as obtained from the PSPS as steers were on similar diets beginning on d 91.

### Sera metabolites

Circulating concentrations of NEFA by dietary treatment through d 97 are presented in Fig. 4. Circulating concentrations of NEFA were not affected (*P* = 0.18) by length of time spent on the receiving diet. However, a treatment × day interaction was observed (*P* < 0.01); concentration of sera NEFA differed on d 42 (*P* < 0.01), whereas steers spending only 28 d on the receiving diet had the least circulating concentration of sera NEFA (0.1660 mEq/L), differing from REC56 and REC84 steers (*P* < 0.01; 0.2472 and 0.2381 mEq/L, respectively). Circulating concentration of NEFA indicates the breakdown of triglyceride stores for energy use as adipose tissue is mobilized to supply metabolic needs of the animal (Bowden 1971). By d 42, REC28 steers had been on a finishing ration for 7 d, and therefore would have had increased energy intake, resulting in greater retained energy compared to REC56 and REC84 steers, who remained on a less energy-dense, receiving ration. However, this response was not sustained throughout the 97-d receiving period among treatments, as sera NEFA did not differ (*P* ≥ 0.23) at any other timepoint. This is likely because of increased DM consumption throughout the receiving period. The difference in DM consumption among treatment groups began to lessen and was not different (*P* = 1.00) throughout the cumulative receiving period, but steers on the REC28 treatment did consume more DM from d 29 to 56 (*P* < 0.01; 7.7 vs 7.0 kg) compared to the REC56 and REC84 steers. Furthermore, steers in the present study were never in a negative energy balance, regardless of treatment, and thus relied on dietary energy intake to maintain energy homeostasis rather than bodily energy stores, thus supporting the lack of differences observed. Robison et al. (2023) also observed minimal differences in circulating concentrations of sera NEFA in newly received beef calves fed 3 different roughage inclusions (15, 30 and 45% prairie hay).

**Figure 4:**
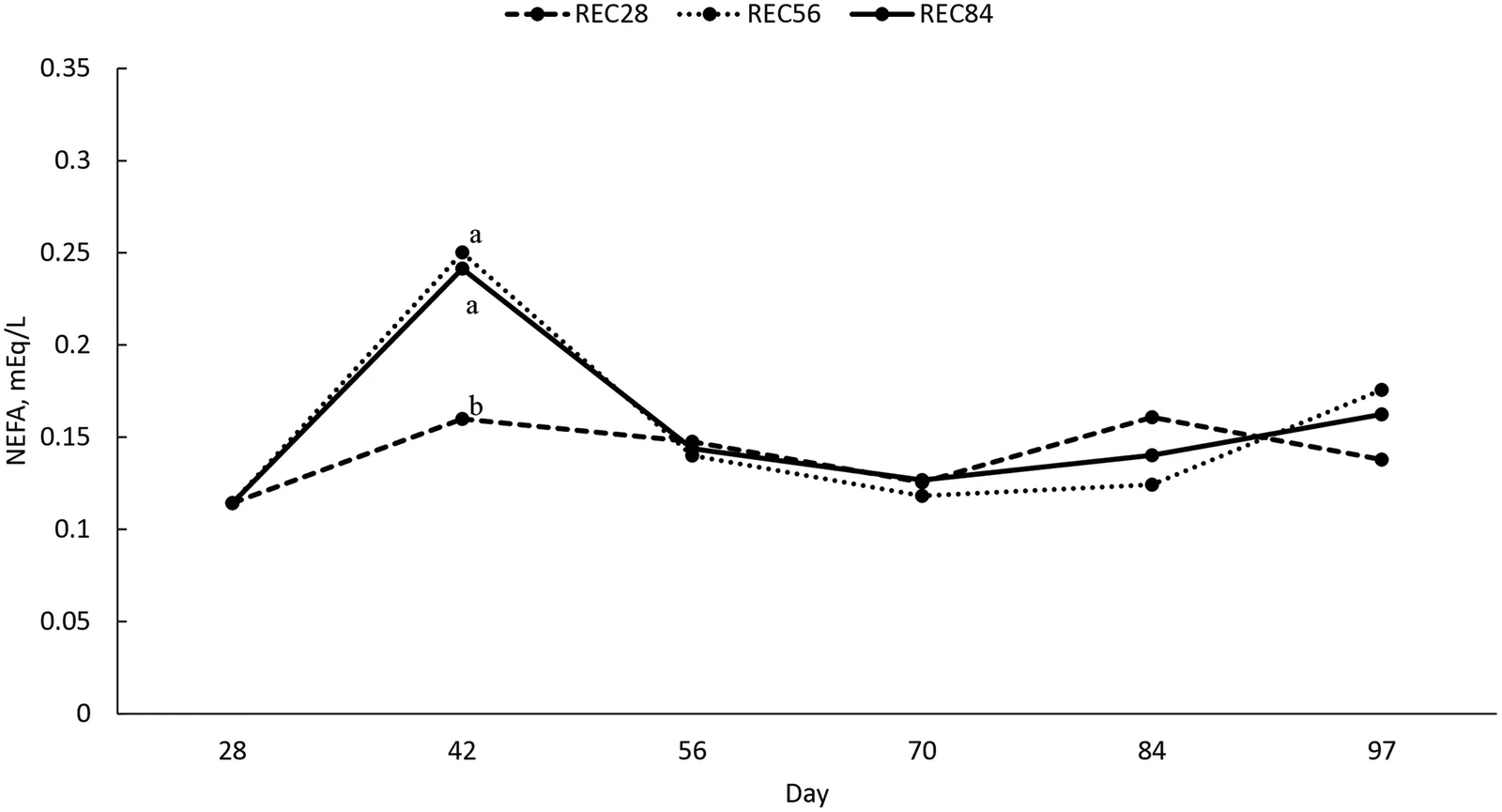
Circulating concentrations of sera non-esterified fatty acids (NEFA) of Angus × Holstein steers fed a receiving diet for either 28 (REC28), 56 (REC56) or 84 d (REC84) post feedlot-arrival. Effect of treatment, *P* = 0.18; day, *P* < 0.01; treatment × day, *P* < 0.01. Standard error of the mean = 0.0153 mEq/L. Concentration of sera NEFA on d 28 was used as a covariate in the model as treatments were not initiated until d 28 post-sampling. ^a,b^Within a day, treatment means without a common superscript differ (*P* ≤ 0.10).

Circulating concentrations of SUN by dietary treatment through d 97 are presented in Fig. 5. A treatment × day interaction was observed (*P* = 0.05). Circulating concentrations of SUN were affected (*P* < 0.01) by length of time spent on the receiving diet and differed (*P* ≤ 0.10) among treatments on d 70 and 84 and were not different (*P* ≥ 0.10) at any other timepoint measured. Overall, steers on the REC84 treatment exhibited the greatest concentration of circulating SUN (12.76 mg/dL) but did not differ from REC28 steers (*P* = 0.37; 12.41 mg/dL) and steers on the REC56 treatment had the least concentration of circulating SUN (11.49 mg/dL), differing from REC84 and REC28 steers (*P* ≤ 0.02). Blood urea nitrogen can be affected by N concentration of the diet, N solubility or degradability of the diet, energy content of the diet, and level of feeding (Hammond 1983). Steers on the REC84 treatment spent the longest time on the 30R diet, not transitioning to the 21R intermediate diet until d 84 and began the 12R diet on d 91. The 30R diet provided the greatest concentration of CP (17.8%), whereas the 12R provided the least (16.7%). Greater dietary CP has been observed to increase blood urea nitrogen in feedlot steers (Vasconcelos et al. 2009). However, Robison et al. (2023) observed no difference in urea nitrogen concentration in newly received calves fed 3 different roughage inclusions (15, 30, and 45%) of prairie hay), reducing in CP as roughage inclusion increased (16.94, 15.96, and 14.98%, respectively). Greater circulating concentration of SUN can be indicative of muscle protein catabolism. However, the authors observed reduced urea nitrogen concentration over time, indicating the time response was likely because of more reliance on dietary N rather than muscle catabolism. The values observed in the present study indicate across all treatments SUN fell well within the recommended values (11 to 15 mg/100 ml) for growing feedlot steers (Hammond 1983), indicating that all steers were in adequate protein status. Therefore, differences in SUN observed in the present study are difficult to explain. Sera glucose by dietary treatment through d 97 are presented in Fig. 6. No treatment effect (*P* = 0.41) or treatment × day interaction were observed (*P* = 0.45). However, an effect of day (*P* < 0.01) was noted for circulating concentrations of sera glucose, where sera glucose concentrations decreased with increasing days on feed. This response was in agreement with Quigley et al. (1991), who also observed decreasing blood glucose levels with increasing time spent on feed in young calves.

**Figure 5:**
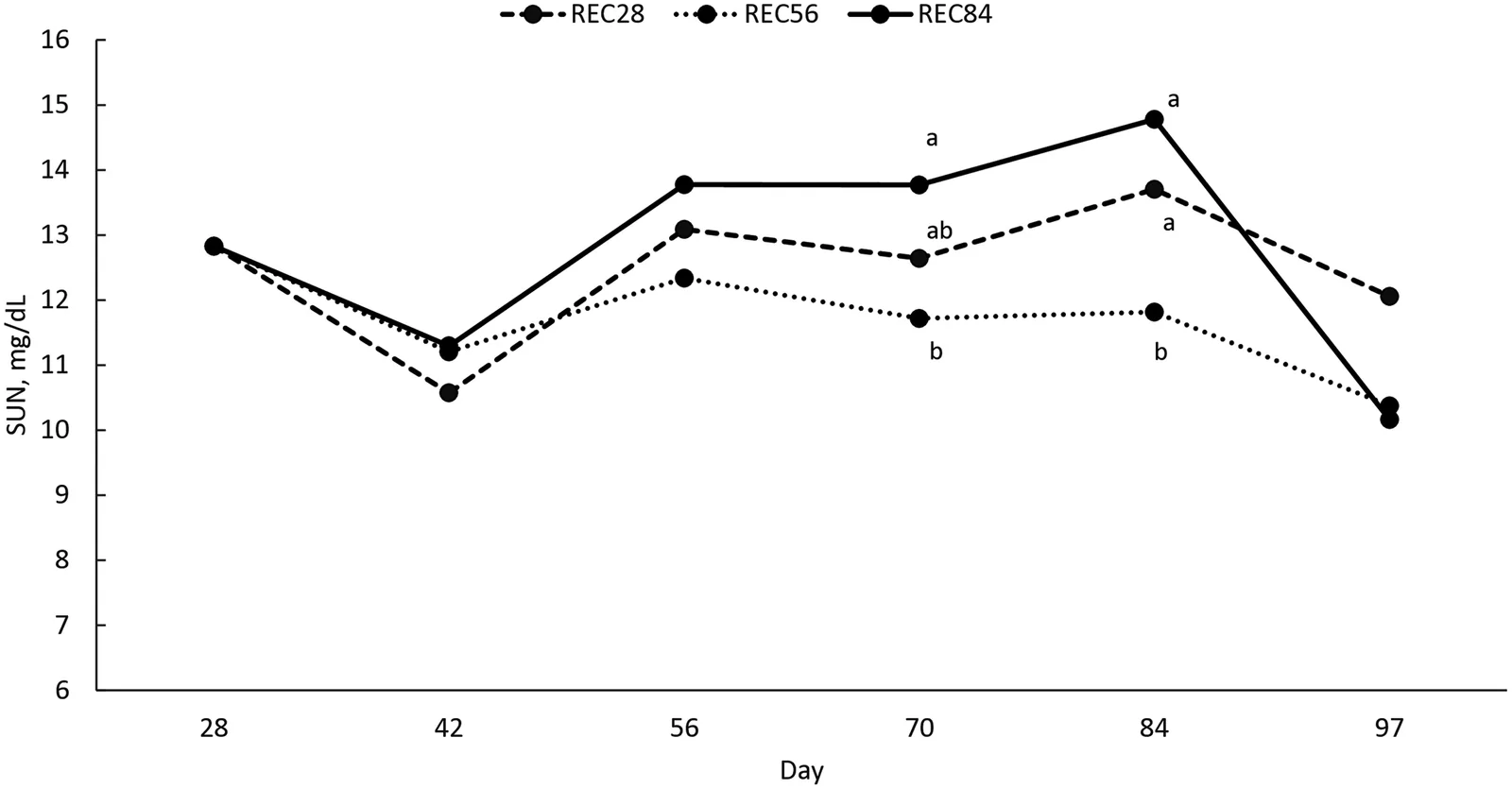
Circulating concentrations of sera urea-nitrogen (SUN) of Angus × Holstein steers fed a receiving diet for either 28 (REC28), 56 (REC56) or 84 d (REC84) post feedlot-arrival. Effect of treatment, *P* < 0.01; day, *P* < 0.01; treatment × day, *P* = 0.05. Standard error of the mean = 0.6255 mg/dL. Concentration of SUN on d 28 was used as a covariate in the model as treatments were not initiated until d 28 post-sampling. ^a,b^Within a day, treatment means without a common superscript differ (*P* ≤ 0.10).

**Figure 6:**
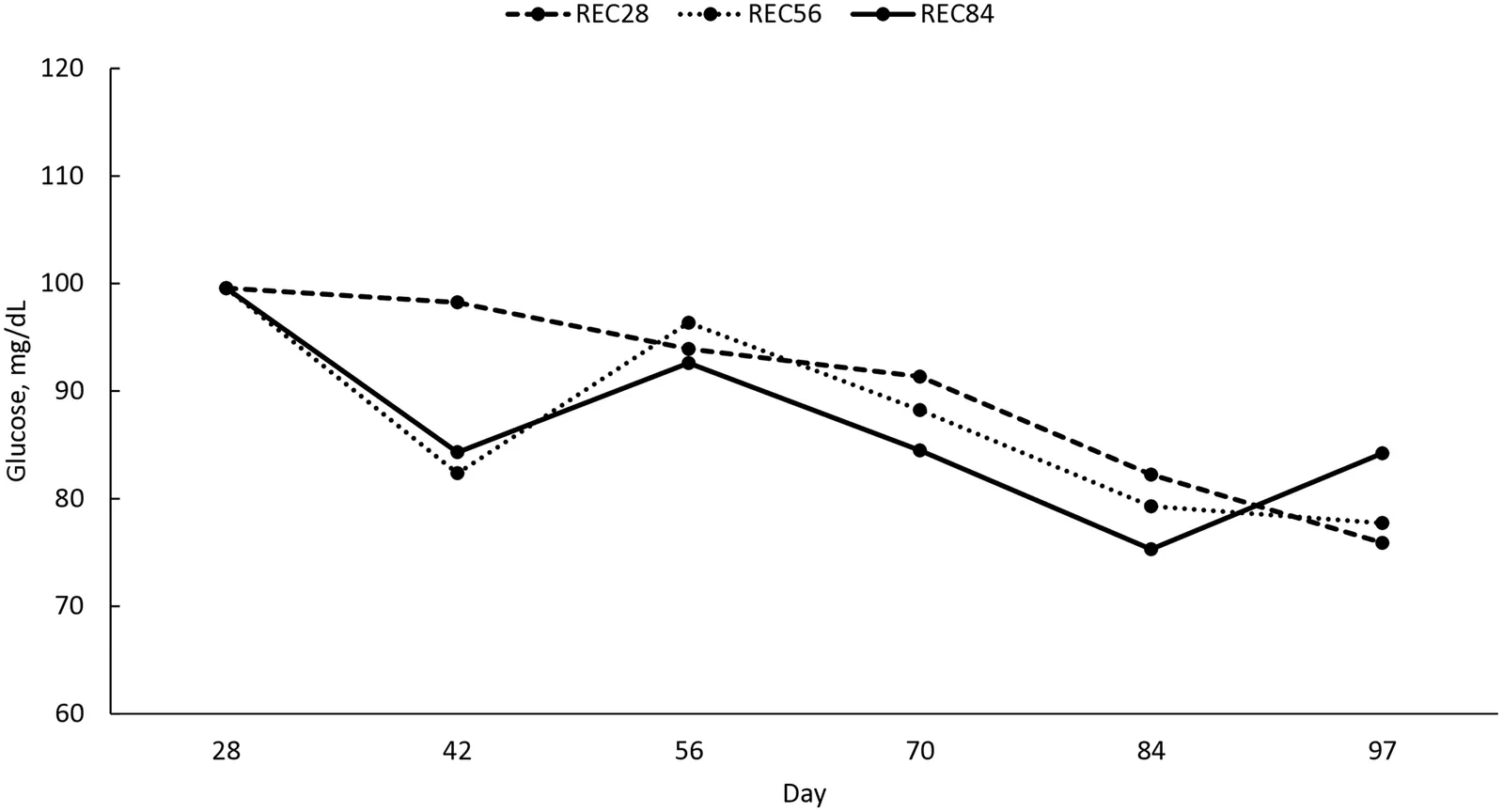
Circulating concentration of sera glucose of Angus × Holstein steers fed a receiving diet for either 28 (REC28), 56 (REC56) or 84 d (REC84) post feedlot-arrival. Effect of treatment, *P* = 0.41; day, *P* < 0.01; treatment × day, *P* = 0.45. Standard error of the mean = 5.08 mg/dL. Concentration of sera glucose on d 28 was used as a covariate in the model as treatments were not initiated until d 28 post-sampling.

## Conclusion

Increasing the length of time Angus × Holstein steers spent on the receiving ration worsened receiving phase growth performance but did not influence overall feeding-period growth performance or carcass characteristics. Rumination activity was positively associated with the length of time spent on the receiving diet. However, when expressed as rumination per kg of NDF and peNDF consumed, rumination activity decreased with increasing time spent on the receiving diet. The results of this study indicate that reducing the time spent on a finishing ration may have resulted in positive improvements in prevalence of liver abscesses. However, numerical reductions in carcass weight and increased use of roughage may not make implementation of this strategy economical. This study provides additional knowledge regarding receiving period management of beef × dairy crossbred steers and further understanding of the complexities of previous management, diet characteristics, roughage type and inclusion, and performance of Angus × Holstein steers.

## Funding

This research was sponsored in part by the Beef Nutrition Program, the National Institute of Food and Agriculture and the South Dakota State University Experiment Station [HATCH- SD00H690-19].

## Conflicts of interest

None declared.

## Data Availability

Data can be made available upon reasonable request to the Corresponding Author (ZKS).

## List of abbreviations

12R: 12% roughage equivalent diet
21R: 21% roughage equivalent diet
30R: 30% roughage equivalent diet
ADF: Acid detergent fiber
ADG: Average daily gain
AFBW: Final body weight adjusted to 28% empty body fat
AOAC: Association of Official Analytical Chemists
BW: Body weight
CP: Crude protein
CV: Coefficient of variation
DM: Dry matter
DMI: Dry matter intake
DOF: Days on feed
DP: Dressing percentage
EBF: Empty body fat
EE: Ether extract
EG: Energy gain
EM: Energy for maintenance
FBW: Final body weight
FMIA: Fluorescent microsphere immunoassay
G:F: Gain-to-feed ratio
HATCH: USDA Hatch Act funding program
HCW: Hot carcass weight
MQ: Maintenance coefficient
N1: Nitrogen
NASEM: National Academies of Sciences, Engineering, and Medicine
NDF: Neutral detergent fiber
NE: Net energy
NEFA: Non-esterified fatty acids
Neg: Net energy for gain
Nem: Net energy for maintenance
O:E: Observed-to-expected ratio
peNDF: Physically effective neutral detergent fiber
PSPS: Penn State Particle Separator
QG: Quality grade
RE: Roughage equivalent
REA: Ribeye area
REC28: Receiving diet fed for 28 days
REC56: Receiving diet fed for 56 days
REC84: Receiving diet fed for 84 days
rNDF: Roughage neutral detergent fiber
SAS: Statistical Analysis System
SD: Standard deviation
SEM: Standard error of the mean
SUN: Sera urea nitrogen
TMR: Total mixed ration
USDA: United States Department of Agriculture
YG: Yield grade
